# Fungal pathogens of plants: deciphering the mechanisms of Fusarium wilt

**DOI:** 10.18699/vjgb-26-62

**Published:** 2026-07

**Authors:** M.P. Bankin, А.А. Samsonova, T.A. Rozhmina, M.G. Samsonova

**Affiliations:** Peter the Great St. Petersburg Polytechnic University, St.Petersburg, Russia; Peter the Great St. Petersburg Polytechnic University, St.Petersburg, Russia; Federal Research Center for Bast Fiber Crops, Torzhok, Russia; Peter the Great St. Petersburg Polytechnic University, St.Petersburg, Russia

**Keywords:** Fusarium oxysporum, host-pathogen interaction, plant immunity, effectors, Fusarium oxysporum, взаимодействие растение–патоген, иммунитет растений, эффекторы

## Abstract

Fusarium oxysporum (Fo) is among the most dangerous soilborne pathogens, causing Fusarium wilt and root rots in over 100 plant species worldwide. Some pathogen strains can also infect immunocompromised animals and humans. Consequently, studying the molecular mechanisms associated with pathogen virulence and the plant immune response at different stages of disease development is of paramount importance. The process of host recognition by the pathogen and all stages of the infection process involve a wide repertoire of specific signaling molecules, effector proteins, receptor complexes, as well as interconnected and overlapping signaling pathways. Plants, in turn, have evolved a complex defense system to counter this attack: they also possess intricate molecular-level mechanisms that, triggered by pathogen assault, transmit signals to activate a defensive response. In this review, we examine the main currently known molecular mechanisms of Fo-host interaction within the plant-pathogen system: from plant detection and directed hyphal growth driven by chemotropism, to complex interactions at the level of immune response and specific fungal tactics for its suppression. The review includes sections dedicated to the dynamics of plant infection, pathogen genome organization and its genomic diversity, plant immune response and pathogen suppression tactics, as well as an analysis of the main known effector molecules of the pathogen and associated transcription factors. Special emphasis is put on the special form of Fo that infects flax (Linum usitatissimum L.).

## Abstract (ru)

Несовершенные грибы вида Fusarium oxysporum (Fo) относятся к числу самых опасных почвенных патогенов, вызывая фузариозное увядание и корневые гнили более чем у 100 видов растений по всему миру. Некоторые штаммы патогена способны также поражать организмы животных и человека с ослабленным иммунитетом. В связи с этим особую важность представляет изучение молекулярных механизмов, ассоциированных с вирулентностью патогена и иммунным ответом растения на разных стадиях развития заболевания. Процесс обнаружения потенциального хозяина патогеном и все стадии развития инфекционного процесса включают в себя широкий репертуар специфических сигнальных молекул, эффекторных белков, рецепторных комплексов, а также взаимосвязанных и перекрывающихся сигнальных путей. Растения, в свою очередь, выработали сложную систему защиты, чтобы противостоять этой атаке: они тоже обладают комплексными механизмами на молекулярном уровне, которые, будучи запущенными в результате атаки патогена, передают сигналы для активации защитного ответа. В данном обзоре мы рассматриваем основные из известных на сегодняшний день молекулярные механизмы взаимодействия Fo с хозяином в системе «растение–патоген»: с момента обнаружения растения и направленного роста гиф патогена, обусловленного реакцией хемотропизма, и до сложных взаимодействий на уровне иммунного ответа и специфических тактик его подавления со стороны гриба. Обзор включает разделы, посвященные динамике заражения растений Fo, рассмотрению особенностей организации генома патогена и его геномного разнообразия, иммуннoму ответу растений и тактике его подавления патогеном, а также анализ основных известных эффекторных молекул патогена и связанных с ними транскрипционных факторов. Отдельное внимание уделено специальной форме Fo, поражающей растения льна (Linum usitatissimum L.).

## Introduction

Fusarium wilt is a dangerous plant disease caused by pathogenic strains of the fungus Fusarium oxysporum (Fo) (Gordon, 2017; Zhang, Ma, 2017). It poses a serious threat to agriculture, and Fo ranks among the top ten most destructive fungal plant pathogens worldwide (Kommedahl et al., 1970; Dean et al., 2012; Rozhmina et al., 2022). Fo infection can lead to catastrophic crop losses, a prime example being Fusarium wilt of banana (Widinugraheni et al., 2018; Zhang et al., 2024). In addition to Fusarium wilt, some Fo strains can also cause root rot (Gargouri Jbir et al., 2024; Ma K. et al., 2024).

The pathogenicity of Fo is host-dependent, as strains infecting one plant species typically do not cause disease in others (Kistler, 1997; O’Donnell et al., 1998; Edel- Herrmann, Lecomte, 2019). Based on this host specificity, pathogenic Fo strains have been classified into so-called formae speciales (ff. spp.), of which over 100 have been described to date (Dean et al., 2012; Edel- Hermann, Lecomte, 2019). Besides infecting plants, forms capable of infecting animals and humans are also known (Zhang et al., 2020). Fo exhibits a polymorphic lifestyle, varying among genotypes, ranging from soil saprophytes and endophytic strains to specialized parasites (Dean et al., 2012). Fo endophytes inhabit tissues of living plants without causing any negative effects on their function and development (de Lamo, Takken, 2020). Another peculiarity is that sexual reproduction has never been observed in Fo, despite the presence of conserved mating-type genes (MAT1-1 or MAT1-2) characteristic of sexually reproducing species (Yun et al., 2000; Fayyaz et al., 2023; Zhang et al., 2024). For instance, most pathogenic Fo strains infecting flax studied by us contained the MAT1-2 mating-type locus idiomorph, corresponding to mating type a, and only one strain contained the MAT1-1 idiomorph, corresponding to mating type alpha (Logachev et al., 2024). Since Fo reproduces asexually, the very fact of the conservation of these genes in the pathogen’s genome remains a puzzle

Host perception and virulence in Fo involve several receptors and signaling cascades. The membrane protein Msb2 regulates the Fmk1 signaling pathway, which plays a key role in Fo virulence (Pérez-Nadales, Di Pietro, 2011). Members of the Velvet complex (LaeA/ VeA/VelB/VelC), Ras proteins, G-protein components, and the cAMP pathway can also regulate Fo virulence (Husaini et al., 2018). The four proteins of the Velvet complex play a critical role in ensuring chromatin accessibility and expression of the beauvericin biosynthesis gene cluster – a depsipeptide mycotoxin. The VeA and LaeA proteins were necessary for full virulence of Fo on tomato plants (López-Berges et al., 2013). The Rho1 GTPase of Fo plays an important role in maintaining hyphal architecture and infecting plants, but not animals (Martínez-Rocha et al., 2008). Genes for the alpha and beta subunits of G-proteins, FGA2 and FGB1, are involved in the pathogenicity of Fo f. sp. cubense. FGA2 directly regulates fungal virulence, wheras FGB1 is involved in both virulence and development through multiple pathways, including the cAMP-PKA pathway (Guo et al., 2016).

## Dynamics of plant infection by Fo

The process of plant colonization by the pathogen begins with the detection of the root by Fo spores or hyphae. Host plant identification is based on chemotropism – the directed growth of the pathogen along the concentration gradient of chemicals secreted by the plant (Nordzieke et al., 2019). Plant root exudates contain carbohydrates that induce germination of chlamydospores and microconidia of the tomato pathogens Fo f. sp. lycopersici (Fol) and Fo f. sp. radicis-lycopersici (Forl) (Steinkeller et al., 2005; Turrà et al., 2015). Additionally, hyphal chemotropism towards tomato roots is triggered by secreted plant peroxidases (Turrà et al., 2015; Nordzieke et al., 2019; Sridhar et al., 2020).

After spore germination, both endophytic and pathogenic Fo strains colonize the plant root system. Fungal hyphae penetrate roots through wounds, cracks in the epidermis, lateral root emergence points, or by direct penetration into the root tip, depending on the Fo strain and plant species (Zhou et al., 2020). Hyphae reach the xylem vessels via the root cortex apoplast. Both pathogenic and non-pathogenic strains colonize the cortex, but although the initial colonization pattern is similar, the extent and nature of colonization differ at later stages (Validov et al., 2010). Typically, only pathogenic strains can efficiently penetrate into the xylem vessels, from which they colonize the aboveground tissues (Mes et al., 2000; Benhamou, Garand, 2001; Van der Does et al., 2019). A possible explanation for this was recently provided by Ayukawa et al. (2021). It was found that colonization of Arabidopsis stems by the Fo f. sp. conglutinans (Focn) strain Cong:1-1 is suppressed by the activity of the CYP79B2/CYP79B3 genes, which control the synthesis of tryptophan-derived secondary metabolites. One Focn chromosome, ChrSC10/SC20, containing the linked genes Six8 and PSE1, enabled stem colonization beyond the root xylem vessels in double mutants of these genes

## Fo genome and genetic diversity

To date, dozens of genomes of many Fo formae speciales infecting economically important crops have been sequenced: e. g., fragariae (Henry et al., 2021), cepae (Armitage et al., 2018), vasinfectum (Seo et al., 2020), ciceris (Fayyaz et al., 2023), pisi (Williams et al., 2016; Jenkins et al., 2021), as well as endophytic strains (Wang B. et al., 2020). All these genomes are compartmentalized and exhibit a mosaic architecture, comprising, on the one hand, gene-rich, transposon-poor regions containing highly conserved housekeeping genes, and on the other hand, gene-poor, repeat-rich regions containing rapidly evolving genes associated with virulence (e. g., effector genes) (Dong et al., 2015). Additional characteristics of the variable part of the Fo genome can include AT-rich isochores, specific chromatin modifications, and physical organization as accessory chromosomes, which enhances genome compartmentalization and facilitates its rapid evolution (Frantzeskakis et al., 2019; Torres et al., 2020).

**Fig. 1.**
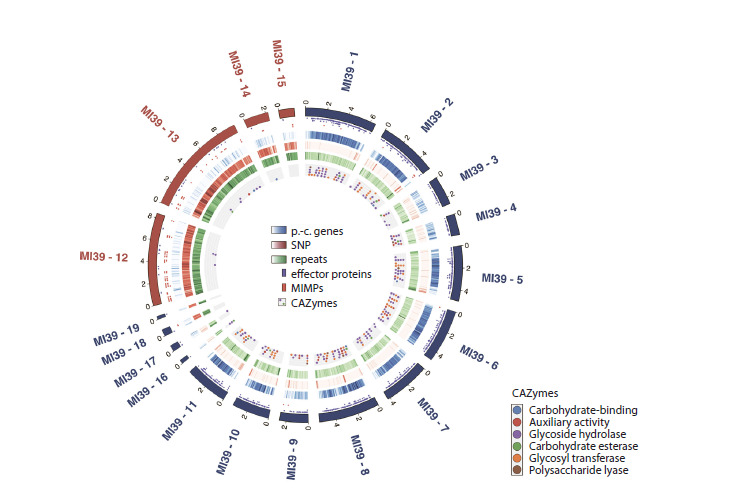
Organization of the Fo f. sp. lini genome. The Folini reference genome was assembled from Illumina short and PacBio long DNA reads of the highly virulent strain MI39 (Samsonova et al., 2021). In the Circos diagram, the outer circle depicts the chromosome ideogram, where chromosomes are assigned to the core (blue) or variable (red) genome compartments. The next two rings (purple and red ticks) show genes encoding effectors (necessary for virulence) and genomic positions of MIMP transposons, respectively. The density of protein-coding genes (p.-c. genes), single nucleotide polymorphisms, and repetitive elements along the chromosomes is represented as blue, red, and green tracks. The color intensity gradient reflects changes in density. The darkest shade corresponds to the maximum density values. The innermost track (grey with circular glyphs) shows the location of enzymes involved in carbohydrate synthesis and degradation (CAZymes). CAZyme types are denoted as follows: blue – carbohydrate-binding, red – auxiliary activity, purple – glycoside hydrolases, green – carbohydrate esterases, orange – glycosyltransferases, brown – polysaccharide lyases.

 Most known Fo genomes have around 11 chromosomes belonging to the core genome. The variable part of the genome can be entirely embedded within core chromosomes as extended blocks, making all chromosomes chimeric (Fo f. sp. cubense) (Zhang et al., 2024), or partially incorporated into chimeric chromosomes and partially represented as separate accessory chromosomes (in Fo f. sp. lycopersici, Fo f. sp. conglutinans) (Ma L.-J. et al., 2010; Wang Y. et al., 2024). There are genomes lacking chimeric chromosomes, such as Fo f. sp. lini (Fig. 1) or the endophytic strain Fo47 (Kanapin et al., 2020; Wang B. et al., 2020; Samsonova et al., 2021). The number of accessory chromosomes varies depending on the strain and forma specialis, ranging from 1 to 9. Accordingly, Fo genome sizes and the number of proteincoding genes vary within 50–70 Mb and 14,000–21,000, respectively (Ma L.-J. et al., 2010; Williams et al., 2016; Armitage et al., 2018; Kanapin et al., 2020; Seo et al., 2020; Wang B. et al., 2020; Samsonova et al., 2021; Fayyaz et al., 2023; Wang Y. et al., 2024).

**Fig. 2.**
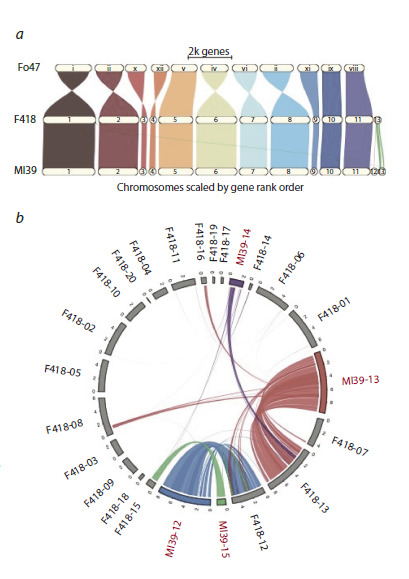
Comparison of the MI39 and F418 genomic assemblies. a, Synteny map for the core genome regions of two Folini isolates MI39 and F418, as well as the endophytic strain Fo47. b, Circos plot (Krzywinski et al., 2009) showing syntenic regions between the accessory chromosomes of these same strains.

Analysis of individual formae speciales genomes revealed high collinearity of core chromosomes and a lack of collinearity between homologous accessory chromosomes (Fig. 2), indicating that accessory chromosomes in individual genomes undergo multiple structural rearrangements (Bates et al., 2024; Logachev et al., 2024). Individual comparisons also demonstrated a high level of variability in the repertoire of individual genes. For example, only 54 % of genes of the entire Folini gene repertoire were present in the genomes of 13 isolates of this forma specialis. Moreover, different Folini isolates differ in their repertoire of genes encoding effectors and other secreted proteins necessary for infection, allowing them to effectively bypass plant defense mechanisms (Logachev et al., 2024).

Phylogenetic analysis of Fo reveals polyphyletic separation, where isolates infecting the same host plant can fall into different clades. For instance, on the maximum likelihood tree based on the EF-1 alpha gene sequence for a group of 50 isolates, including 13 Folini genomes and genomes of other formae speciales, isolates infecting flax are found in different clades (Logachev et al., 2024). This means they evolved independently on different genetic backgrounds during evolution. Since Fo lacks sexual reproduction, the mechanism for this was elusive for a long time, but it was recently shown that horizontal chromosome transfer is responsible for the emergence of new pathogenic lineages.

Horizontal transfer of accessory or lineage-specific chromosomes has been described in several plantpathogenic filamentous fungi (Vlaardingerbroek et al., 2016). The possibility of horizontal chromosome transfer in Fo was first demonstrated for the tomato-infecting forma specialis, Fol. As a result of co-incubation of microconidia from two strains – the pathogenic strain Fol007 and the endophytic strain Fo47, differing in antibiotic resistance, – strains resistant to both antibiotics were selected (Ma L.-J. et al., 2010). These strains were pathogenic when infecting tomato, and contour-clamped homogeneous electric field electrophoresis showed they all contained, besides chromosomes from the endophytic strain Fo47, one or two new chromosomes. One of these chromosomes corresponds to accessory chromosome 14 of Fol. Horizontal chromosome transfer has now also been demonstrated for another Fo forma specialis: radicis cucumerinum (Forc), pathogenic on cucumber, melon, and watermelon (Vlaardingerbroek et al., 2016; van Dam et al., 2017; Li et al., 2020).

## Mechanisms of plant immune response

Plant defense mechanisms include several types of immune responses (Jones, Dangl, 2006; Kourelis, van der Hoorn, 2018). The first, called PTI (Pattern-Triggered Immunity), is activated by Pathogen-Associated Molecular Patterns (PAMPs) or Damage-Associated Molecular Patterns (DAMPs), which are typically small molecules produced by the pathogen or resulting from plant cell wall destruction (Balint-Kurti, 2019). PAMPs are recognized by Pattern Recognition Receptors (PRRs) (Kunstler et al., 2016), which are membrane-bound proteins. In response, several signaling events occur, such as activation of the MAP kinase cascade, Ca2+ influx into the cytosol, and production of Reactive Oxygen Species (ROS). Consequently, triggering a series of signaling reactions leads to the synthesis of antimicrobial compounds and activation of defense genes

The second line of defense is initiated by effectors – proteins that can modify cellular targets to suppress PTI (Tsuda, Katagiri, 2010). Effector-Triggered Immunity (ETI) is activated when a plant R-gene protein recognizes an effector encoded by a pathogen avirulence (Avr) gene (Kunstler et al., 2016). This type of interaction, the so-called “gene-for-gene” relationship, was first discovered by Flor (Flor, 1971) when studying the flax/flax rust host-pathogen interaction. Effector interactions with R-gene products elicit a strong defense response called the Hypersensitive Response (HR), often culminating in localized cell death. PTI and ETI are mutually linked and together potentiate the immune response (Ngou et al., 2021; Nguyen et al., 2021).

Proven intracellular functions of fungal effectors include manipulation of metabolic processes or transcriptional regulators (Djamei et al., 2011; Plett et al., 2014; Tanaka et al., 2014; Weßling et al., 2014). It was recently shown that fungal effectors can also suppress intracellular PTI signaling (Di et al., 2017; Irieda et al., 2019; Navarrete et al., 2022), although the generality of this mechanism for fungi remains to be proven (Tintor et al., 2020).

## Tactics of plant immune response suppression by Fo

**Fig. 3.**
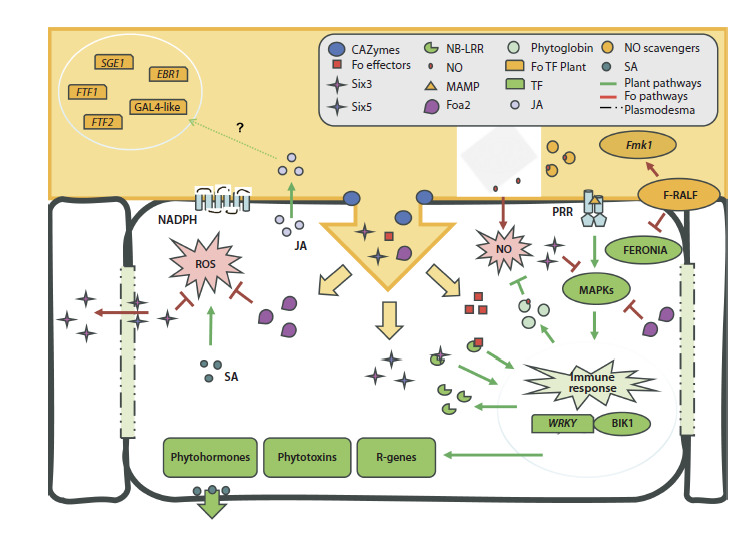
Schematic representation of Fo effector interaction with key components of the plant immune system. In the figure, red lines indicate the main pathways of pathogen action on the plant, green lines indicate pathways of the plant’s response to pathogen invasion. Abbreviations in the legend: CAZymes – enzymes actively degrading and synthesizing carbohydrates; Six3/Six5 – small, cysteine-rich Fo effector proteins; NB-LRR/PRR – cytoplasmic and transmembrane proteins encoded by R genes; NO – nitric oxide; MAMP – microbe-associated molecular patterns; Foa2 – Fo effector; phytoglobins – plant proteins involved in NO inactivation; Fo TF Plant – Fo transcription factors; TF – transcription factors; JA – jasmonic acid; NO scavengers – Fo proteins involved in NO inactivation; SA – salicylic acid.

Recently, many new Fo effector genes have been identified, and several generalizations have been made regarding their genomic location, presence of their products in the plant, and their mechanism of action (Fig. 3). First and foremost, it became evident that effector genes are located not only on accessory chromosomes (Sun et al., 2022). Studies of Folini genomes showed that most effector genes localize to the core part of the genome and that about 40 % of such genes are not present in the genomes of all strains. This means different Folini isolates have different effector repertoires (Logachev et al., 2024).

SIX proteins are the most well-studied class of effectors in Fo. These small, cysteine-rich proteins were isolated from the xylem sap of tomato seedlings infected with Fo f. sp. lycopersici (Rep et al., 2002). SIX genes belong to 14 gene families and are located on variable chromosomes. Each forma specialis has a characteristic profile of SIX proteins and gene sequences (De Sain, Rep, 2015). Endophytic Fo strains usually have fewer predicted effector genes than pathogenic strains (van Dam et al., 2017; de Lamo, Takken, 2020; Constantin et al., 2021). Interestingly, in our studies, we were unable to detect SIX genes in two low-virulence Folini strains, F365 and F482 (Logachev et al., 2024). Likely, Folini isolates can infect plants even in the absence of SIX genes. Nevertheless, other Folini genomes, regardless of their pathogenicity status, displayed identical sets of SIX gene families, namely SIX1, SIX7, SIX10, SIX12, and SIX13. Thus, it can be assumed that the SIX gene repertoire is conserved in the flax-infecting forma specialis (Logachev et al., 2024).

Loss of SIX genes reduces Fo pathogenicity. Such results have been obtained for genes SIX1, SIX3, SIX5, SIX6, and SIX8, primarily in the tomato-infecting Fo f. sp. lycopersici, and for the SIX6 gene in Forc, the cucumber-infecting forma specialis (Rep et al., 2004; Houterman et al., 2009; Gawehns et al., 2014, 2015; Ma et al., 2015; Ayukawa et al., 2021).

Despite their apparent importance for host plant infection, the functions of most SIX proteins remain unknown (Jangir et al., 2021). The only exception is the SIX3 (AVR2) gene (Houterman et al., 2009), which manipulates plant immunity at the PTI level (Fig. 3). As a virulence factor, Avr2 suppresses several PTI responses, including ROS accumulation, callose deposition, and MAPK activation. Recent studies in Arabidopsis thaliana established that the mechanism of Avr2 action involves the receptor-like cytoplasmic kinase BOTRYTISINDUCED KINASE1 (BIK1). Bacterial flagellin (and its derivative, flg22 peptide) and fungal chitin are two well-studied PAMPs recognized by Pattern Recognition Receptors (PRRs) of A. thaliana – FLAGELLIN SENSING 2 (FLS2) and CHITIN ELICITOR RECEPTOR KINASE 1 (CERK1)/LYSINE MOTIF RECEPTOR KINASE 5 (LYK5), respectively. Upon binding flg22, FLS2 forms a signaling complex with its co-receptor BAK1. BAK1 is a leucine-rich repeat receptor kinase. Within the FLS2-BAK1 complex, BAK1 phosphorylates BIK1, a receptor-like cytoplasmic kinase, at several sites (Lin et al., 2014). BIK1 is then monoubiquitinated by E3 ligases RHA3A and RHA3B, allowing it to dissociate from the FLS2-BAK1 complex into the cytosol (Blekemolen et al., 2023).

The immunoregulatory function of BIK1 depends on both nuclear and cytosolic localization. Nuclear localization of BIK1 is necessary for activating defense gene expression, e. g. by phosphorylating WRKY transcription factors involved in regulating defense hormones – salicylic and jasmonic acids. Cytoplasmic localization of BIK1 upon dissociation from the immune receptor complex on the plasma membrane is necessary, for example, for triggering ROS-mediated signaling through phosphorylation of the RBOHD complex localized on the plasma membrane (Fu et al., 2024). Avr2 disrupts monoubiquitination of BIK1, and since monoubiquitination of BIK1 is necessary for its internalization, in the presence of Avr2, BIK1 remains on the plasma membrane and cannot perform its immunoregulatory function (Blekemolen et al., 2023; Li et al., 2024).

In the tomato pathogen, the effector genes Six3 and Six5 share a common promoter located upstream of their coding regions. It was shown that Six3 and Six5 localize to both the cytoplasm and nucleus of cells, but their colocalization is detected only at plasmodesmata (Cao et al., 2018). The interaction of Six5 with Six3 alters the physical properties of plasmodesmata, allowing Six3 to move into neighboring cells. In a susceptible plant, this benefits the pathogen and promotes infection spread.

Besides receptor-like kinases, targets of Fo effectors at the first level of defense can be the MAP kinase signaling cascade and oxidative stress (Fig. 3). It was shown (Tintor et al., 2020) that stable expression of the effector gene Foa2 from Fo5176, infecting A. thaliana, in cells of this plant blocks accumulation of both ROS and phosphorylated MAP kinases in response to PAMP stimulation.

In Fo, nitric oxide (NO) is a key signaling molecule involved in the interaction of the fungus with the host plant (Terrón-Camero et al., 2023) (Fig. 3). Fusarium oxysporum f. sp. cubense (Foc) race R1 of the bananainfecting forma specialis devastated trade in Gros Michel bananas (Musa acuminata), and now the tropical race TR4 threatens global production of its replacement – Cavendish bananas (Viljoen et al., 2020). Upon infection with race TR4, plants showed unique induction of genes localized in mitochondria involved in nitric oxide (NO) biosynthesis and detoxification pathways (Zhang et al., 2024). Methyl jasmonate stimulation led to a NO burst in TR4 isolates. Knocking out two highly inducible TR4 genes involved in the NO biosynthesis pathway significantly reduced NO production and fungal virulence, indicating that inducing the NO burst directly affects TR4 infectivity. It was suggested that the ability to produce the NO burst, which simultaneously disarms host defense and protects the fungus from toxic environmental impact, was acquired by race TR4 through the emergence of race-specific genes on accessory chromosomal regions that participate in NO production and fungal virulence.

F-RALF proteins are also effectors that suppress host immunity by alkalinizing the environment surrounding plant roots (Fig. 3). During plant infection, Fo causes an increase in extracellular pH, leading to activation of the MAPK signaling cascade Fmk1, thereby promoting invasive hyphal growth and virulence (Masachis et al., 2016). Mutants of Fo f. sp. lycopersici in the FOXG_21151 gene, encoding an F-RALF protein with pH-raising function, showed reduced ability to colonize the host plant and caused significantly less mortality than the wild type or complemented strain, as well as activation of plant defense reactions (induction of defense genes, accumulation of reactive oxygen species, and callose deposition). In tomato plants, F-RALF action is mediated by interaction with the receptor-like kinase FERONIA, which also interacts with endogenous plant RALF proteins and functions as a negative regulator of immune response (Stegmann et al., 2017).

Some effectors are highly conserved and apparently present in all fungi interacting with plants (Redkar et al., 2022a). Deletion of genes for such effectors, named ERC (Effectors Required for Colonization), in the genome of the pathogenic tomato fungus Fo4787 reduces its virulence and leads to rapid activation of immune response genes, and in the genome of the endophytic strain Fo47 it reduces root colonization extent and ability to act as a biocontrol agent. ERC effectors are also involved in infection of the liverwort Marchantia polymorpha by strain Fol4287.

Besides effectors, genes encoding secondary metabolites (Ito et al., 2004; Coleman et al., 2011), lipid metabolism (He, Dung, 2020) and CAZymes (Carbohydrate- Active enZymes) (Menna et al., 2021), as well as genes controlling fungal penetration (Liu et al., 2019) also play a role in Fo virulence.

## Transcription factors and effector activity

The transcription factor SGE1 (SIX Gene Expression 1) regulates expression of SIX genes in Fol in vivo (Michielse et al., 2009) (Fig. 3). In other Fo formae speciales, SGE1 is necessary for SIX gene expression, as well as for genes, the products of which are associated with secondary metabolism (Jonkers et al., 2012; Brown et al., 2014; Zhao S. et al., 2020). The SGE1 gene is an ortholog of the conserved fungal transcription factor Wor1 from Candida albicans and Histoplasma capsulatum, which regulates morphological transition and is associated with virulence towards humans (Zhao S. et al., 2020).

In addition to SGE1, located in the core genome, a group of genes named FTF (Fusarium Transcription Factor) (Fig. 3) is found on both core and accessory chromosomes of Fol. In Fo f. sp. phaseoli, expression of FTF1 increases during infection of bean plants and is necessary for fungi pathogenicity (de Vega-Bartol et al., 2011). Knockout of FTF genes suggested they regulate pathogenicity mainly by controlling effector expression (Niño-Sánchez et al., 2015). Expression profile analysis showed that transcript levels of SGE1 and FTF1 increase during infection processes, and constitutive expression of FTF1, FTF2, or SGE1 induces expression of a large overlapping set of known effector genes in Fol, indicating interaction between these transcription factors (van der Does et al., 2016).

Another transcription factor in Fo, EBR1 (Enhanced Branching 1), is involved in virulence, similar to its ortholog in F. graminearum (Zhao C. et al., 2011; Jonkers et al., 2014). EBR1 is located on chromosome 7 of Fo f. sp. lycopersici and is thus part of the core genome, while other EBR paralogs are located on accessory chromosomes. EBR1 mutants showed slower growth in culture and reduced virulence against tomato plants (Niño-Sánchez et al., 2015).

## Promising methods for combating Fusarium infection

The development of Fo control strategies is complicated by the fact that different isolates of the same forma specialis may use different mechanisms to suppress host immunity (Logachev et al., 2024), as well as by the high evolutionary rate of the accessory genome compartment, allowing pathogens to easily and quickly bypass developed defense mechanisms

Among modern methods of combating Fusarium wilt, special attention should be paid to biological control methods as the most environmentally friendly, as well as to the use of disease prediction models based on analysis of microbial community composition characteristic of specific soils.

The soil microbiome represents one of the most complex systems directly linked to plant development and health (Raaijmakers, Mazzola, 2016). Bacterial and fungal communities have the ability to influence hormonal signaling in plants, thereby manipulating defense mechanisms (Eichmann et al., 2021), as well as agricultural productivity, forming key ecosystem functions such as nutrient cycling and resistance to plant pathogens (Yuan et al., 2020).

These properties of microbial communities can be used as means to combat Fusarium wilt. For instance, Bubici et al. (2019) demonstrated the possibility of controlling Fusarium wilt in field conditions with efficacy up to 79 % using Pseudomonas spp. strains and up to 70 % using several endophytes and Trichoderma spp. strains. Lower biocontrol efficacy (42–55 %) was observed with arbuscular mycorrhizal fungi, Bacillus spp., and non-pathogenic Fusarium strains (Bubici et al., 2019).

Use of the EGB strain of the myxobacterium Corallococcus sp. reduced cucumber Fusarium wilt incidence by 9.6 % in greenhouse conditions, and by 66.0 and 53.9 % in field conditions in 2015 and 2016, respectively (Ye et al., 2020). Meanwhile, the rhizosphere microbiota formed in a hydroponic system with multiple parallel mineralization was able to control Fo population dynamics but not eliminate the fungal pathogen. The surviving Fo in the hydroponic system formed chlamydospores upon contact with the rhizosphere microbiota. The authors suggested that the microbiota suppresses Fo spread by controlling pathogen morphogenesis and creating an ecosystem allowing coexistence with Fo (Fujiwara et al., 2013). Maximum reduction in pea root rot severity (80 %) under greenhouse conditions was achieved by a synergistic triple treatment consisting of arbuscular mycorrhizal fungi, Trichoderma harzianum, and Pseudomonas fluorescens (El-Sharkawy et al., 2021).

Application of arbuscular mycorrhiza and T. harzianum on tomato plants also revealed their effectiveness in mitigating Fusarium wilt by 45.14 and 44.91 %, respectively, compared to untreated infected plants (Meddad-Hamza et al., 2023). P. fluorescens mitigates peanut root rot symptoms even under field conditions and significantly inhibits Fo growth (Ren et al., 2024). Studies on the interaction of Fo f. sp. lycopersici with tomato plants showed that fusaric acid production by the pathogen causes systemic changes in the rhizosphere microbiota and directly influences the recruitment of specific disease-suppressive taxa (Jin et al., 2024).

To date, the most well-known ways to limit the risk of flax Fusarium wilt are the creation of resistant cultivars and suitable crop rotations. However, the emergence of new pathogenic strains requires the development of alternative methods to reduce disease, primarily biological control methods. For example, Planchon et al. (2021) discovered a Bacillus subtilis strain possessing biocontrol activity. Using thermogravimetry, the authors showed that this strain, acting cultivar-specifically, promoted the strengthening of stem cell walls.

Mathematical models can be used to predict the potential risk of Fusarium outbreak by identifying key biological indicators and features characteristic of the microbiome of diseased soil. For instance, machine learning can classify Fo-infected and non-infected soil samples based on their bacterial and fungal communities (Yuan et al., 2020). It turned out that the healthy soil microbiome has a higher abundance of Streptomyces mirabilis, Bradyrhizobiaceae, Comamonadaceae, Mortierella, and non-pathogenic Fusarium strains. The random forest method identified 45 bacterial and 40 fungal OTUs that classified soil health status with over 80 % accuracy (Yuan et al., 2020).

## Conclusion

The lack of effective methods to combat Fusarium wilt threatens the production of economically important crops worldwide, including flax. Upon infection, Fo penetrates plants directly through the roots via infectious hyphae and then colonizes and spreads within the xylem vessels (Yadeta, Thomma, 2013). Perception of a potential host in Fo begins with directed chemotropic growth towards the plant roots (Nordzieke et al., 2019), involving both root exudates inducing germination of Fo chlamydospores and microconidia (Steinkeller et al., 2005; Turrà et al., 2015), and secreted plant peroxidases (Turrà et al., 2015; Nordzieke et al., 2019; Sridhar et al., 2020). Many molecular aspects of this phenomenon require further study, for example, concerning the functions and interactions of receptors involved in the early stages of infection (Jiang et al., 2019).

During co-evolution with their hosts, phytopathogens have developed molecular mechanisms allowing them to effectively overcome plant immune responses. To date, significant material has accumulated on signaling cascades activated in response to Fo infection (Fig. 3). However, our knowledge of the regulation of this pathogen’s interaction with the plant is limited to transcriptome response analysis (van der Does et al., 2016), and the role of DNA methylation and other epigenetic mechanisms in this process remains to be investigated.

A recent meta-analysis of plant transcriptional response to Fo infection (Cai et al., 2022) identified hub genes encoding several CAZymes, including xyloglucanase and β-glucosidase. Further research is needed for a deeper understanding of molecular mechanisms in Fo concerning carbohydrate metabolism and the enzymes involved. As part of our work, it was shown that carbohydrate-degrading proteins constitute a significant part of the secretome of Folini isolates (Logachev et al., 2024).

It is evident that genes encoding important virulence factors are regulated by more than one transcription factor; for example, FTF1 and SGE1 jointly participate in regulating SIX family genes (van der Does et al., 2016). SIX genes are specifically expressed during infection, but exactly how FTF1 and SGE1 promote SIX gene expression during infection is still unknown; thus, a detailed functional analysis of these important virulenceassociated transcription factors is necessary.

The question of mechanisms determining host specificity of different Fo formae speciales deserves special attention. At first glance, it might seem that since there are many effector proteins, the control of this process should be polygenic. However, it turned out this is not the case (Li et al., 2020). For example, strain Forc016 Fo f. sp. radicis-cucumerinum can cause disease in cucumber, melon, and watermelon, while strain Fom005 Fo f. sp. melonis (Fom) can only infect melon plants. Forc016 was transformed with the effector gene g14035, located on the pathogenic chromosome of Fom005. Transformants had greatly reduced or absent pathogenicity towards cucumber while retaining pathogenicity towards melon and watermelon. This suggests that the protein encoded by g14035 is recognized by an immune receptor in cucumber plants.

Another result indicating that host switching can be determined by a single gene was obtained by transforming the flax-infecting forma specialis with the FoPDA1 gene from Fo f. sp. pisi. This gene encodes a demethylase that detoxifies the phytoalexin pisatin in pea plants (Coleman et al., 2011).

Endophytic interactions of Fo with plants are much more common than pathogenic ones (de Lamo, Takken, 2020; de Lamo et al., 2021; Redkar et al., 2022b), yet such interactions are much less studied at the molecular level, and the genetic basis underlying endophytic and pathogenic behavior is unknown.

Currently, no biocontrol agent provides a 100 % level of protection against the harmful effects of Fo. The same picture was observed in breeding resistant agricultural cultivars: 100 % resistance is usually provided by a combination of genes. So, improvements of biological protection tools should also move towards creating communities including several bacteria/fungi, each reducing the harmful effect of Fo. This imperative, however, elevates the problem of developing biocontrol agents to a new level of complexity, as it requires accounting for and analyzing interactions within a complex multicomponent system composed of community members, the plant, the pathogen, other rhizosphere microbes, and the environment.

Thus, despite the significant current knowledge on the molecular mechanisms of Fo interaction with plants, numerous questions requiring further study remain.

## Conflict of interest

The authors declare no conflict of interest.
